# Machine learning model from a Spanish cohort for prediction of SARS-COV-2 mortality risk and critical patients

**DOI:** 10.1038/s41598-022-09613-y

**Published:** 2022-04-06

**Authors:** Alejandro Reina Reina, José M. Barrera, Bernardo Valdivieso, María-Eugenia Gas, Alejandro Maté, Juan C. Trujillo

**Affiliations:** 1 Lucentia Department of Software and Computing Systems, University of Alicante, Carretera San Vicente del Raspeig s/n, 03690 Alicante, Spain; 2 Lucentia Lab, Av. Pintor Pérez Gil, 16, 03540 Alicante, Spain; 3 The University and Polytechnic La Fe Hospital of Valencia, Avenida Fernando Abril Martorell, 106 Torre H 7a planta, 46026 Valencia, Spain

**Keywords:** Diagnosis, Disease prevention, Public health, Computer science, Scientific data, Statistics, Classification and taxonomy, Data integration, Data mining, Data processing, Machine learning, Predictive medicine

## Abstract

Patients affected by SARS-COV-2 have collapsed healthcare systems around the world. Consequently, different challenges arise regarding the prediction of hospital needs, optimization of resources, diagnostic triage tools and patient evolution, as well as tools that allow us to analyze which are the factors that determine the severity of patients. Currently, it is widely accepted that one of the problems since the pandemic appeared was to detect (i) who patients were about to need Intensive Care Unit (ICU) and (ii) who ones were about not overcome the disease. These critical patients collapsed Hospitals to the point that many surgeries around the world had to be cancelled. Therefore, the aim of this paper is to provide a Machine Learning (ML) model that helps us to prevent when a patient is about to be critical. Although we are in the era of data, regarding the SARS-COV-2 patients, there are currently few tools and solutions that help medical professionals to predict the evolution of patients in order to improve their treatment and the needs of critical resources at hospitals. Moreover, most of these tools have been created from small populations and/or Chinese populations, which carries a high risk of bias. In this paper, we present a model, based on ML techniques, based on 5378 Spanish patients’ data from which a quality cohort of 1201 was extracted to train the model. Our model is capable of predicting the probability of death of patients with SARS-COV-2 based on age, sex and comorbidities of the patient. It also allows what-if analysis, with the inclusion of comorbidities that the patient may develop during the SARS-COV-2 infection. For the training of the model, we have followed an agnostic approach. We explored all the active comorbidities during the SARS-COV-2 infection of the patients with the objective that the model weights the effect of each comorbidity on the patient’s evolution according to the data available. The model has been validated by using stratified cross-validation with k = 5 to prevent class imbalance. We obtained robust results, presenting a high hit rate, with 84.16% accuracy, 83.33% sensitivity, and an Area Under the Curve (AUC) of 0.871. The main advantage of our model, in addition to its high success rate, is that it can be used with medical records in order to predict their diagnosis, allowing the critical population to be identified in advance. Furthermore, it uses the International Classification of Diseases, Ninth Revision, Clinical Modification (ICD 9-CM) standard. In this sense, we should also emphasize that those hospitals using other encodings can add an intermediate layer business to business (B2B) with the aim of making transformations to the same international format.

## Introduction

The outbreak of the SARS-COV-2 pandemic has led to a disruptive change in society throughout the world at all levels. The health problems derived from the infection pose a challenge for the scientific community, since the knowledge associated with the disease is very limited. In this sense, the scientific community has focused its efforts on looking for solutions, vaccines, and palliatives of the pandemic, trying to accelerate the process of returning to normality^1^.

The rapid evolution of the pandemic, together with the unknown clinical characteristics of the disease, has posed a challenge for the health area. The pandemic has generated problems related to the use of hospital resources, the unexpected evolution of patients or the choice of the most appropriate treatment, taking into account the clinical status that patients already had prior to the disease^2^.

The increase in the availability of data in the health area allows the application of Big Data analytics and Artificial Intelligence (AI) techniques^3,4^. Various studies in *state-of-art* literature^5^ present its advantages and applicability in different areas such as Decision Support System to improve the allocation of resources in health management^6^ or clinic and prognostic models for the prediction of various diseases such as cancer^7^ or heart disease^8,9^. The advantages of these techniques can also be indirectly reflected in the increase in scientific publications related to the topic^10^, providing various benefits such as helping to provide better care and reducing costs^11^. These results show the success of these techniques in the health field, being able to discover relevant clinical information hidden in a large amount of data regardless of the format^12–14^ (image, text, or raw data), which plays a key role when clinical decision must be taken. More specifically, AI techniques allow us to automate processes and quickly analyze the results as long as there are sufficient data available. This is key to converting data into information that allows us to quickly react to critical cases such as the SARS-CoV-2 virus. In addition, with the appearance of new strains^15^, such as the Alpha (United Kingdom, Sep-2020), Beta (South Africa, May-2020), Gamma (Brazil, Nov-2020), Delta (India, Oct-2020), the most recent Omicron (Multiple countries, Nov-2021), or others that have yet to appear which may vary in their effects, it is essential to be able to train specific models for specific diseases as soon as data is available.

However, some studies^16–18^ are based on statistical techniques. These techniques have been shown to be imprecise as the volume of information increases^19,20^. To overcome these problems, the AI techniques allow to analyze the large number of variables present and their impact on critical patients.

Regarding AI techniques, we can find two approaches: Deep Learning (DL) and Machine Learning (ML) approaches. Considering DL approaches, there are previous works with good results^21,22^. However, DL techniques present problems or challenges of model explainability. Although there are studies that cover this problem by using techniques such as SHAP^23,24^, or in image classification model by visualising convolutional filters, the interpretability of DL models is still a hot topic^25^. We should point out here that one of main goals of this paper is to provide a clear set of variables that influence the evolution of patients. For this reason, we propose an interpretable and explainable ML model. In out ML model, we can manage its explainability by setting the weight of each variable in the model, which allows us to validate and extract insights of which variables most influence in the evolution of patients.

According to ML, in a recent systematic review of ML models constructed to predict the evolution of the disease in patients or the risk of mortality in patients^2^, authors concluded that, out of the studies analysed in the review, many were conducted by using only data from Chinese patients. This carries a risk of bias and may raise questions about the applicability and accuracy of existing ML prediction models in other populations of patients who can be potentially different. Therefore, the objective of the study presented in this paper is to build and validate a ML model for patients infected by SARS-CoV-2 and to provide information on a cohort of Spanish patients. We believe that different ML models on different patients from different nations are absolutely needed. This would set the basis for ulterior research comparing and validating the evolution of patients from different nations and taking into consideration particular variables of the different races. Clearly, this study is out of the scope of this paper. This is the main reason why there are more and more studies on different patient nationalities.

Other studies^26–29^ were carried out in the first months of the pandemic. Thus, the number of samples covered is small because they use data collected during 3 months in the best case for the construction of the models. Incorporating a greater number of samples allows the population used for training to approach a Gaussian normality. This allows us to draw more robust conclusions and capture the different intrinsic casuistic in any population. In this sense, our study is more robust in terms of the number of patients included, since it uses data from patients affected by the infection for approximately 8 months.

We can also find studies based on symptoms^17,29–35^ such as headache, vomiting, fever, shortness of breath, diarrhea, muscular soreness, and other variables as comorbidities. Symptom variables are normally obtained in primary care and stored as handwritten notes and non-tabulated information. Our approach obtains similar results and does not depend on variables that are usually collected in textbook format. Moreover, our model uses structured information and quality variables in standard format in a way that facilitates its integration with the hospital information systems.

Furthermore, in recent literature it can be read papers where authors reduce the number of characteristics of the algorithms by applying feature selection techniques^28,30–32,34^ or domain knowledge^17^. Although in general terms these techniques improve the precision of the algorithms by eliminating noise^36^, in cases such as SARS-CoV-2 that involve complex casuistic it is difficult to determine exactly if the noise is real data that affects the problem studied. Infrequent combinations in the data set can be considered an anomaly although they do have an implication on the outcome. This implies that information is lost. Given that we are faced with a new problem where much information is unknown, we follow an agnostic approach where we use all the available comorbidities in order to explore the importance of each comorbidity.

Moreover, it can be found studies that present the problem of grouping together different diseases^26^ such as cancer or respiratory problems. Currently, there are more than 100 different types of cancer. Thus, our hypothesis is that different cancer diseases will interact with respiratory effects caused by SARS-COV-2 differently. Similarly, we assume that some respiratory diseases will interact with SARS-COV-2 in a more severe way. For this reason, we do not group diseases into their families. Instead, we explore them individually to know their impact on the evolution of patients.

Thus, the main goal of this paper is to present a ML model and a case study on a cohort of Spanish patients (n = 5378). The data have been obtained during 8 months of the pandemic, from February 27, 2020 to November 12, 2020. Our ML model is based on the medical records for detecting the probability of death of patients with SARS-COV-2 based on age, sex and comorbidities recorded in the ICD-9 format. One of the main remarks of our paper is that the provided model accurately predicts the probability that a patient dies during her infection. We have also used regularization techniques in order to avoid overfitting. Furthermore, another key advantage of our model, is that it allows what-if analysis with the inclusion of comorbidities that can appear during the infection. This allows the early detection of future and potential critical cases and consequently, the more severe effects in SARS-CoV-2 infected patients can be mitigated by taking preemptive actions.

The rest of the paper is structured as follows: First, the Method and methodology section is presented. Within this section, we describe all the different methodological steps applied to our case study. These steps can be summarized as (i) the regulation under the method was applied and the approval by the corresponding ethical committee, (ii) the description of the data sets and features, (iii) the pre-processing of data, (iv) the explainability of missing values, (v) the ML model training, and finally, (vi) the ML model interpretation and explainability. Afterwards, the “Results” section presents the statistics on the study cohort, the results obtained by the ML algorithms and their optimization, as well as the feature importance obtained by the model. Then, the “Discussion” section discusses the advantages of our proposal and the limitations with different state-of-the-art studies presented in this “Intoduction” section. Finally, the “Conclusion and future works” section summarizes the contribution, results and future challenges.

## Methods and methodology

All methods were carried out in accordance with relevant guidelines and regulations. The study was approved on 3rd June 2020 by the relevant legal and ethics boards, including the committee of ethic for biomedical research with medicines of the University and Polytechnic La Fe Hospital (CEIm La Fe) of Valencia with registration number #2020-181-1. This organization complies with GCP standards (CPMP/ICH/135/95) and with current legislation that regulates its operation also declaring that there is no conflict of interest in the evaluation and authorization of the clinical study, declaring that forementioned project is conforms to ethical regulations on biomedical research with human subjects and is viable in terms of the scientific approach, objectives, material, and methods, etc., described in the application. This is a retrospective study in which the national and international regulations regarding the treatment of health data for secondary purposes have been respected. The legitimacy for the processing of personal data is based on the anonymised or pseudonymised processing of data without consent under the terms provided under Spanish law for in article 16.3 of Law 41/2002, of 14 November, the basic law regulating patient autonomy and rights and obligations regarding clinical information and documentation in relation to the second paragraph of the seventeenth additional provision on the processing of health data of Organic Law 3/2018, of 5 December, on the Protection of Personal Data and guarantee of digital rights.

### Data source and baseline characteristics

The University and Polytechnic La Fe Hospital of Valencia is the reference clinical setting of the La Fe Health Department, a geographical district that covers a population of around 300,000 inhabitants, and it includes two specialties centers and twenty primary care centers. The Electronic Health Care Record of the Hospital has access to data from both primary and specialized care.

La Fe Health Department has deployed an EHR at different care levels, including over 20 million records, effectively organized reaching stage 6 in the eight-stage (0–7) EMRAM maturity model. Currently, the data lake layer includes structured and semi-structured information, coming from several information systems involving clinical activity, such as emergency care settings, outpatient, hospitalization, clinical reports, surgical unit, intensive care unit, hospital at home care. La Fe Health Department has developed a Real-World Data analysis platform composed by the aggregation of 22 datamarts and comprises 750 millions of rows, 84 tables, 4.064 columns.

This study is a retrospective, observational single centre study which includes all individuals undergoing a SARS-CoV-2 test at the Department of Health Valencia La Fe between 27th February 2020 to 12th November 2020, meeting all the inclusion criteria and none of the exclusion. Inclusion criteria: Patients attended at the University and Polytechnic La Fe Hospital of Valencia with a confirmed diagnosis of COVID-19 by RT-qPCR. Exclusion criteria: Patients from whom there were not enough data to be able to make any useful assessment and patients referred to or treated at the hospital with no suspicion of COVID-19 infection. Several studies have reported the importance of age and comorbidities^37,38^ or the sex difference in immune response^39^ in the evolution of patients affected by SARS-COV-2. Therefore, for cases with at least one positive test, we extracted data from the EHR system, including demographics, comorbidities (ICD9 and ICD-10 coding system) and outpatient data.

### Data processing

**Figure 1:**
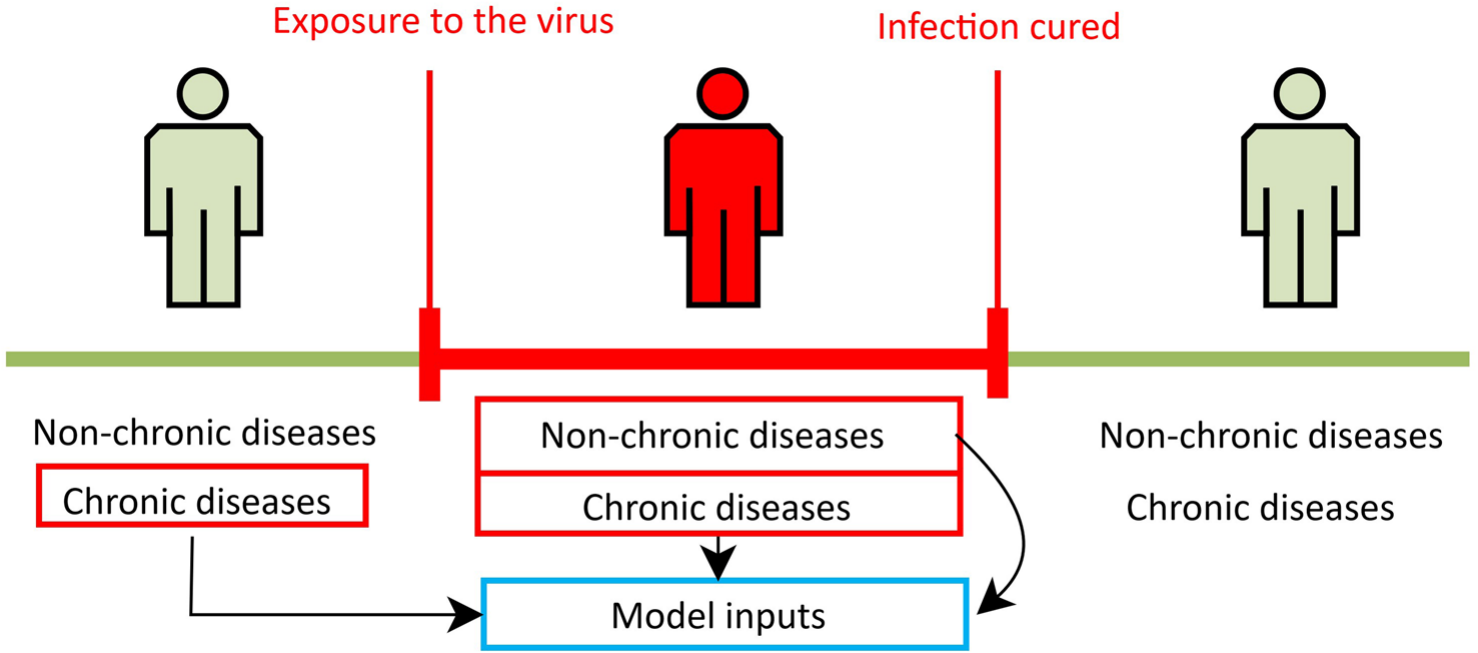
Selection window of the comorbidities. The red window is the period comprised between the beginning of the infection and when the seroconversion occurs.

First, processing is carried out to restrict the data to the time window of the patient’s infection period (Fig. 1). That is, from the time the patient becomes infected until the moment when seroconversion occurs (IgM and IgG immunoglobulins are negative and positive, respectively). The determination of this time window is of utmost importance. It allows us to select the period in which a patient test positive on a molecular test for SARS-COV-2 and confirms infection as well as the moment when the viral load is very low and not detected in the patient. In this case, the comorbidities that appear after this period are not the objectives of this study.

**Figure 2:**
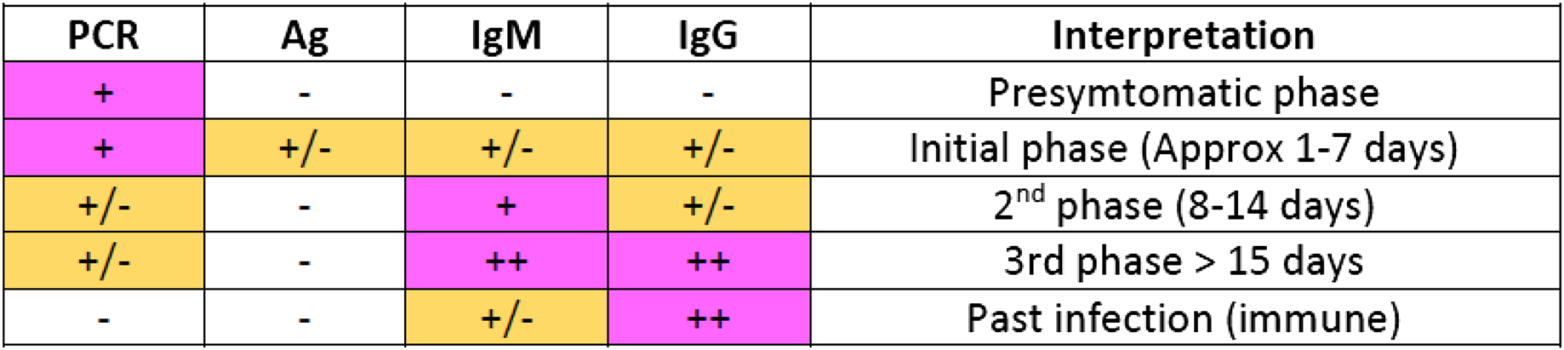
SARS-CoV test interpretation table where we can see if a test can be positive depending on the phase in which the patient is (Instituto de Salud Carlos III).

**Figure 3:**
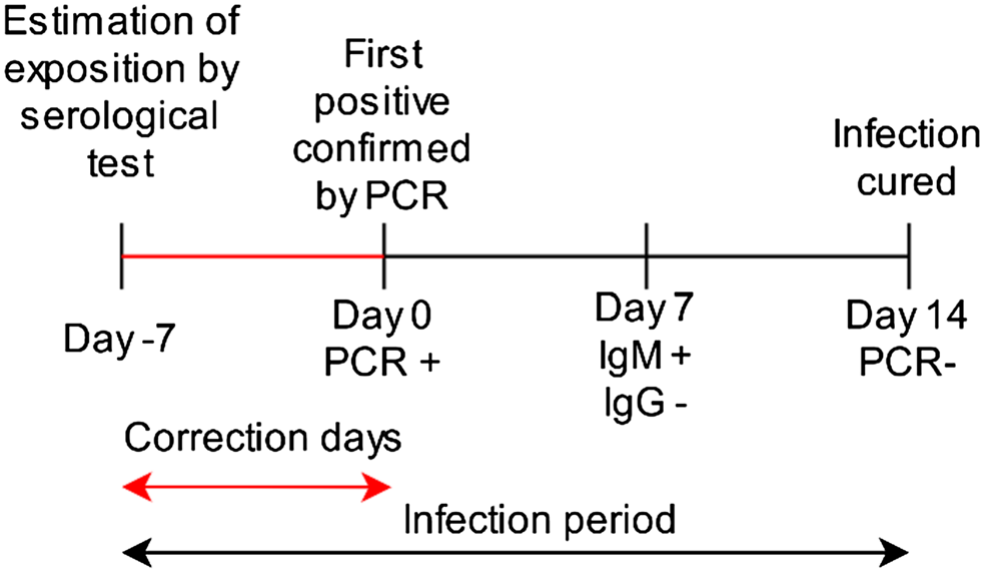
Estimation of the patient exposure to the virus according to traceability of test.

**Figure 4:**
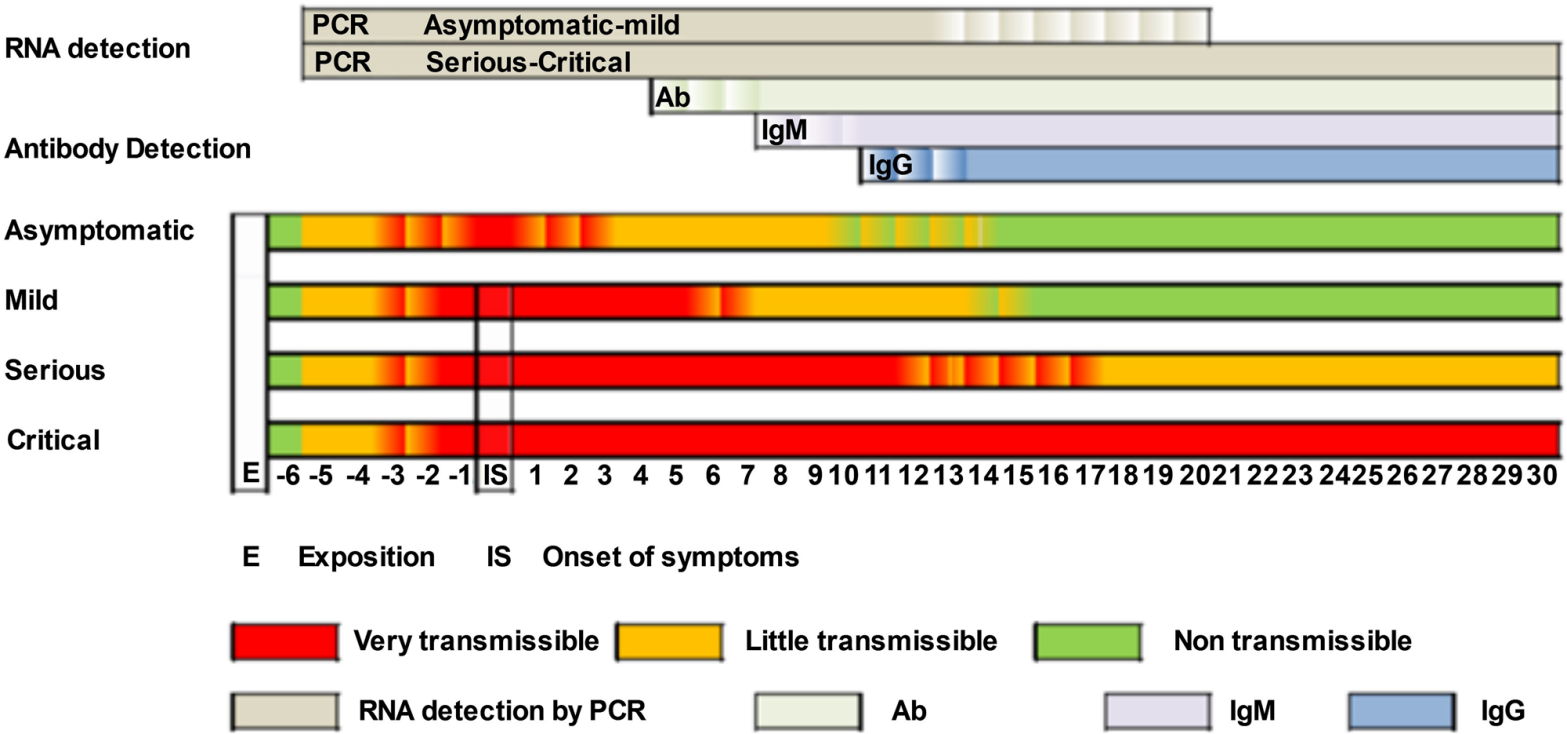
Detection periods of SARS-CoV-2 RNA by PCR and antibodies by serological techniques. On the X axis we can see the number of days that have elapsed, with day 0 being the onset of symptoms. The white stripe of letter “E” indicates the date of exposure to the virus and the beginning of the infection (Instituto de Salud Carlos III).

To limit the data to the specified period, the following steps are performed:

1. We process the results of all tests performed on a patient in the same day. A total of 25,229 patient outcomes are found in the dataset. For example, we can have a patient’s result for PCR and for immunological test result like Ag, IgM, and IgG (Fig. 2).
2. A positive coronavirus test only indicates that the patient is currently infected. Consequently, comorbidities present between the date of exposure to the virus and the confirmation of the first positive should be taken into account. In order to reduce noise in data and approximate as accurately as possible the date of the patient’s exposure to the virus, we applied a correction factor on the date of onset of infection according to the type of positive test (see Fig. 3). In Fig. 4 we can see the detection period of SARS-CoV-2 RNA by PCR and antibodies by serological techniques. In Table 1 we can see a summary of the correction of days applied based on the parameter measured by each one of the tests present in the dataset.
3. The comorbidities of the patient noted by the doctors during the period of the patient’s infection are selected, as well as the chronic morbidities that the patient had previously in his medical history (see Fig. 1).
4. Comorbidities are coded to a single standard ICD-9-CM^40,41^ that allows the integration of the model with the hospital information systems. Comorbidities in ICD-10-CM was mapped to ICD-9-CM format using eCIEMaps v.3.3.8^42^. Some morbidities included in the coding are eliminated because they are directly related to the dependent variable to be predicted. For example, ICDs associated with morbidities such as admission for palliative treatment or brain death imply imminent death. These ICDs would not be available beforehand or do not provide relevant information once the ICD appears.

**Table 1:** Correction factor (days) by kind of test, since a patient may be infected by the virus until a test can detect in general terms that the patient is positive.

Test	Days since positive
PCR	2
IgA	2
Ac	11
IgM	14
IgG	18

### Missing values

After processing the traceability of the tests in the study dataset, we found patients whom we cannot determine the end of the infection. This is due to who recommendation published on 27 May 2020 in which a criterion was established to end the patient’s isolation (of transmission-related precautions) without requiring retesting^43^. This implies that there are patients without a negative test indicating the end of the infection. Considering that the WHO criteria also include patients presenting symptoms, it is not possible to determine and distinguish the end of infection and which comorbidities appear after the end of infection without introducing noise. With the aim of avoiding the inclusion of noise, we discard these patients to finally have a quality dataset with 1201 patients from which we can determine the period of onset and end of infection.

### Model training

For the experimentation we use different machine learning algorithms such as SVM^44^, Logistic regression^45^, K-Neighbors^46^, Decision Tree^47^, Gaussian Naive Bayes^48^, MLP^49^, and ensemble methods like Adaboost^50^ and Bagging techniques^51^. They were implemented in the open-source Python library for machine learning, Scikit-learn^52,53^. All methods were evaluated using a stratified k-fold cross-validation with k = 5 and partitions 80/20^54^. This approach allows subdividing the data set into different sets to avoid overfitting^55^ as well as testing with a different data set than the one used for training. This simulates a real and empirical environment for testing the model^56^.

For the normalization and scaling of numerical variables such as age, we tested with different normalizers and how they affected the results. One-hot-encoding was used for categorical variables such as gender and a multilabel binarizer for the different comorbidities of the patients during infection. Thanks to the computational capabilities available, we have not required to apply dimensionality reduction over the data, thus preserving all the information as input to the machine learning models.

To obtain the algorithm that provides the best result to the problem, we compare different state of the art algorithms. The comparison is carried out using stratified k-fold cross validation, measuring the accuracy of the different models trained with different scalers for numerical variables. This approach allows us to obtain robust results by balancing the classes so that they have the same weight and the results obtained are not affected by the imbalance present in the data. Futhermore, we have also performed Cochran’s Q test to compare the classification accuracies between the different ML techniques.

Once we have found the best algorithm for the use case, we tune the hyperparameters using Particle Swarm optimization (PSO)^57,58^ for algorithm parameter tuning. For this optimization, a stratified k-fold cross-validation with k = 5 and 80/20 partitions are used where the weighted measure of accuracy and sensitivity is obtained in order to reduce false negatives (FN). This case is paramount when predicting that a person will not die when they do. This could imply that less care is offered to the patient than necessary or that the future resources that will be needed are underestimated. Including sensitivity allows us obtaining an algorithm more robust reducing the FN, that is, when the algorithm indicates that the person does not die when in fact the patient dies at the cost of losing precision in the false positives (FP). In other words when the algorithm indicates that the person dies when they do not. This last case is less important because the consequences would be to monitor a patient who is considered critical, which effectively thanks to the care the severe state is avoided.

To analyze the final model obtained and extract insight from its results, the variables that provides more information to the algorithm when making the prediction are analyzed.

### Model interpretation

To verify and interpret the final model obtained in order to know whether results of the presented model are consistent with the existing knowledge of the illness. We analyze the coefficients of the variables obtained by the model.

Taking into account that the building model is logistic regression and the dependent variable of the model is binary (the patient survives or not), its value is a linear combination of the independent variables. Thus, the probability of the dependent variable is modeled as (1)

1 $$logit \left(p\right)= {b}_{0}+{b}_{1}{X}_{1}+{b}_{2}{X}_{2}+\dots +{b}_{n}{X}_{n}.$$

More specifically, considering that the variable to predict is binary and *p* is the probability of prediction to be 1 (patients exitus) we can define the logit $\ell$ or log-odds as the probability of an event happening divided by the probability of that event not happening as shows in (2)

2 $$\ell=\mathrm{log}\left(\frac{p}{1-p}\right).$$

## Results

In this section we will analyze the patient cohort used for the construction of the model, as well as the different results obtained by the different models, their optimization, and the interpretation of the final model obtained.

### Patients

After processing the data, the study includes a cohort of 1201 patients for whom the period of infection could be determined and who were positive for SARS-COV-2 from Feb 26, 2020 to Nov 11, 2020. Women were more represented than men (55.12% vs 44.88%). Mean age was 49.53 ± 24.90 years. Most of the patients presented at least one comorbidity (88.84%), and over half had more than two comorbidities (78.35%). Main comorbidity was Other specified viral infection (33.47%), followed by Essential hypertension (29.14%), Pneumonia (18.82%) and hyperlipidemia (17.23%). 102 patients (8.49%) were transferred to ICU and 154 patients (12.82%) died. Table 2 shows the demographic and comorbidities statistics of the study group.

**Table 2:** Baseline characteristics and comorbidity of patients with coronavirus disease (SARS-COV-2).

		Mean	Std	Min–max
Age of SARS-COV-2 survivors	General N = 1201	49.53	24.90	0–101
	Women N = 662	49.98	25.53	0–101
	Men N = 539	48.97	24.12	0–101
Age of non SARS-COV-2 survivors	General N = 154	80.89	13.11	11–101
	Women N = 75	83.43	14.46	11–101
	Men N = 79	78.49	11.27	49–101
Number of comorbidities in general population	General N = 10,677	8.89	9.83	0–77
	Women N = 5112	8.40	9.05	0–77
	Men N = 5565	13.42	13.57	0–66
Number of comorbidities in the deceased population	General N = 3007	19.52	11.58	0–63
	Women N = 1241	16.77	9.03	1–54
	Men N = 1766	22.35	4	4–63

### Machine learning results

**Figure 5:**
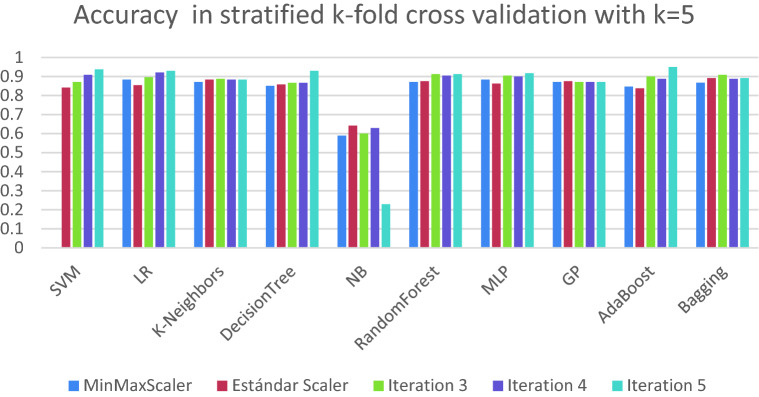
Accuracy obtained in each of the iterations of stratified k-fold cross validation with k = 5 on each of the algorithms used to obtain the best algorithm that works best a priori for the use case.

The preliminary results of the models built are shown in Table 3. In this table, we can see the average result of the algorithms for the stratified k-fold cross validation based on the precision obtained. We can see that due to the small number of numerical variables in the data set, there is not much difference between the results depending on the scaler. In general terms, standard scaler is the one that offers the best result. In Fig. 5, we show the results obtained for each of the algorithms in the different iterations of the stratified k-fold cross validation. The results in general terms are robust since there is little variability between the different scores, which shows that different algorithms can obtain similar acceptable results.

**Table 3:** Matrix of accuracy results, according to different scales and algorithms.

Scaler	SVM	LR	K-neighbors	Decision Tree	Naive Bayes	Random Forest	MLP	GP	AdaBoost	Bagging
MinMax	0.8826	0.8876	0.8759	0.8776	0.5378	0.8901	0.8901	0.8718	0.886	0.8793
Standard	0.8868	0.8968	0.8818	0.8801	0.5378	0.8976	0.8926	0.8718	0.8843	0.8859
MaxAbs	0.8826	0.8876	0.8759	0.8751	0.5378	0.8943	0.8909	0.8718	0.8851	0.8793
Robust	0.8826	0.8968	0.8784	0.8743	0.5378	0.8868	0.8934	0.8726	0.8835	0.8818
Quant-Normal	0.8859	0.8968	0.8843	0.8826	0.5378	0.8951	0.8993	0.8734	0.8843	0.8918
Quant-Uniform	0.8809	0.8876	0.8759	0.8693	0.5378	0.8984	0.8893	0.8718	0.886	0.8793
PowerTransf-yeoJhonson	0.8818	0.8968	0.8776	0.8693	0.5378	0.8935	0.8951	0.8718	0.8843	0.8859

We have realized a Cochran’s Q test with a significance level of α = 0.01 on each stratified k-fold cross validation iteration using standard scaler. The results in Table S2 shows that differences exist between the classification accuracies of ML techniques. As Fig. 5 show, NB present a poor performance (between 30 and 70%) than the rest of algorithms in terms of accuracy. For this reason, we have excluded NB with the aim of comparing the rest of ML techniques. The results indicate in Table S3, with a significance level of α = 0.01, that there are no significative differences between the classification accuracies of the different ML techniques. It must be highlighted that according to the statistical analysis performed, the different algorithms are not significant different.

Despite this, we have chosen logistic regression as the algorithm to optimize due to the interpretability of the model, which is crucial for the medical domain, even though Random Forest gets a slightly better mean result. Random Forest is a set of 100 trees (according to training parameters), where each of the trees uses a subset of variables with its own importance. On the other hand, Logistic Regression has practically the same performance in terms of accuracy. Moreover, due to the nature of the algorithm itself, it allows us to obtain the probability of the event based on the evidence, as well as the coefficients of the variables involved in the decision function. This justifies its selection as the best algorithm. Its coefficients can be directly represented as probabilities with the aim of reducing the gap between machine learning and the medical domain.

**Figure 6:**
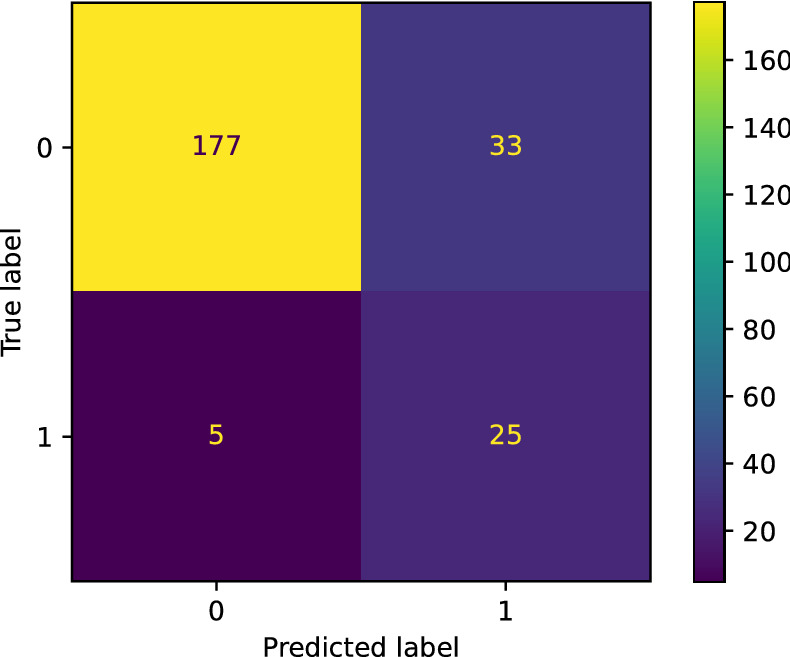
Confusion matrix obtained after optimizing parameters using data not previously seen by the algorithm for the validation of the results. In this case, label 0 means that the patient is alive after infection, whereas 1 means that the patient dies.

For the hyperparameter optimization of the model, the PSO optimization algorithm is used with the weighted measure of accuracy and sensitivity obtained from applying stratified k-fold cross-validation with k = 5, as it has been described earlier in the methodology section. The best result is an accuracy of 84.16%, a sensitivity (also knowns as recall) of 83.33%, a precision of 56.90%, specificity of 84.29% and f1-score of 67.62%. In Fig. 6 we can see the confusion matrix of the model, where its result coincides with the median of the k-fold results. Considering that the true case is that the patient dies, we have a ratio of FN of 16% compared to 26.6% of FN obtained by the model before optimization.

**Figure 7:**
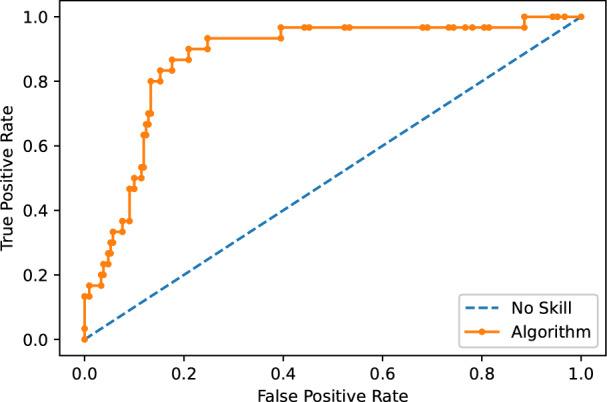
Area under the curve of the best algorithm obtained. The Y-axis show the relation of True Positive (TP) rate and the X-axis show False Positive (FP) rate with AUC = 0.871.

In addition, in Fig. 7 we can see how the final model reaches a good prediction performance in terms of curve ROC (AUC = 0.871).

### Interpretation of the model

Once the final model has been optimised and selected, we can go into more detail on verify and interpret the model in order to know whether results of the presented model are consistent with the existing knowledge of the illness. To this aim, we will analyze the coefficients of the variables obtained by the model as specified in the method and methodology section.

The model finds that the most important variables are Chronic airway obstruction which increases the probability of dead in a 575%. Other variables as Age increase the probability in 145% each 10 year of patients or Acute respiratory failure with a increase of 513%.

As we can see in Supplementary Table S1, among the most influential comorbidities we can find those expected by medical professionals as leading to more severe. However, there are also other unexpected comorbidities as well as some that apparently would be completely unrelated. The explanation of these comorbidities appearing is due to their association with other chronic problems. As the model learns from the underlying sample, patients who suffer for example from dementia show larger health deterioration, which in practice leads to more difficulties to survive to the SARS-CoV-2 infection.

## Discussion

Our study shows that machine learning models are capable of predicting with a high degree of precision the evolution of patients in terms of mortality using demographic variables and patient comorbidities during SARS-CoV-2 infection. For a given patient passed as input, the model obtained can also indicate the probability of the expected outcome, as well as identify and report those comorbidities that have not been taken into account for the result shown (they were not present in the training of the model). This provides more contextual information for medical professionals using the model, allowing them to make more efficient use of medical resources and help them reduce the mortality of patients infected by SARS-CoV-2.

If we compare our results with the literature, we find several studies^28,30,32,34^ that obtain similar or superior results in terms of sensitivity, accuracy and AUC but in which a smaller number of patients are considered. In Ref.^28^ they used a cohort (n = 162), which implies that our approach is more robust since it has been validated on a larger cohort of patients. According to this study^34^, in addition to a larger cohort of patients, we used a data sample to validate the model formed by patients that the model had not previously seen in its training, simulating a real environment^20,30,32,34^, that obtain similar or superior results in terms of sensitivity, accuracy and AUC but in which a smaller number of patients are used.

On the other hand, we have studies^17,18,30,31^ that obtain similar results but with a less specific approach. Consequently, that implies less detail in the patient’s situation. In Ref.^17^ they realized a classification as severe that encompasses different states of patients (the intensive care unit, mechanic invasive ventilation, or death) while our model is more specific in terms of using and knowing which are the morbidities that most affect the mortality of patients. In Ref.^18^ they use a statistical analysis. In our approach we pre-process the data to eliminate data contamination. We also use a different data set than the one used in the construction of the model to verify the model and the conclusions drawn. Regarding Refs.^30,31^ our approach considers a greater number of morbidities, and we offer a broader approach based on different diseases instead of different symptoms.

Finally, we have studies^27,33,35^ that follow an approach like ours, but obtain worse results in terms of AUC. In Ref.^35^, they obtain a result of 0.742 that is still present in the model replication in Ref.^27^ on a new cohort of patients. Our approach improves results mainly in the detection of patients who are going to die, reaching a sensitivity of 83.33% in this case. In addition, our approach allows us to know which are the characteristics that most affect the mortality of patients^19,33,35^, that follow an approach like ours, but obtain worse results in terms of AUC.

Despite these advantages, the current study has limitations. First, for the training of the model, laboratory data were not available, which could improve the results obtained, as well as expand the criteria available for the model in the detection of patients who will need intensive care. Second, we found unrepresentative comorbidities. Consequently, a threshold could be established with the aim of more aggressively eliminate comorbidities that do not reach such threshold. Finally, despite using a much larger cohort of patients than other studies and the good results obtained, we consider that it is still a population belonging to a relatively small geographical area. This could also be solved by using more data from patients from other countries and/or health systems to further validate the robustness of the results.

Despite the limitations, we show that machine learning techniques can play a key role in this type of problem. Among the strengths of the study, we can highlight that this study has used a relatively large cohort of patients compared to other studies in the literature. Furthermore, we have taken special care in data cleaning in order to eliminate noise and using data from patients who have tested positive for SARS-COV-2. Furthermore, our study has used a diverse cohort of patients to whom different lifestyles and different health states can be attributed, obtaining robust results in terms of accuracy and sensitivity. These results have been validated using cross validation techniques, verifying that the chosen model obtains solid results, and using different sets of patients for training and testing. In addition, we follow an agnostic approach where a large set of comorbidities were considered in order to identify which are the morbidities that lead to negative patient evolution.

On the other hand, since our model is based on the international code of ICD 9-CM diseases, it allows the almost integration of the information systems of the hospitals that use it. Alternatively, codes can be converted from other standards to this encoding adding an intermediate layer business to business (B2B), thus being able to quickly integrate it into the hospital systems and reducing data required to be input by users to obtain a prediction.

Finally, our model not only indicates the evolution of the patient but also indicates the probability that this event will occur, providing more contextual information to health workers. This allows the early detection of the most critical patients and consequently, an early intervention which implies a potential reduction in the mortality of patients with SARS-COV-2, as well as a more efficient use of medical resources.

## Conclusion and future works

This study has been developed based on the hypothesis that pre-existing conditions (comorbidities) of patients can increase the severity of patients due to SARS-COV-2. Therefore, we have followed an agnostic approach and developed a machine learning model capable of predict the mortality of patients infected by SARS-COV-2 with robust performance in terms of (AUC = 0.871), accuracy of 84.16% and a sensitivity of 83.33%.

Regarding data sources, we have used a data set (n = 5378) of anonymized patients where we consider demographic variables such as age and sex, as well as data from the medical records of the patients. The model has been validated by using stratified k-fold cross-validation with k = 5, where robust results are obtained for different iterations. The presented model not only offers a high degree of precision, but also offers the probability that the cited event will occur and reports on those comorbidities that were not present in the training of the model. This allows physicians a more efficient use of medical resources, as well as the early detection of the most critical patients allowing an early intervention that potentially reduces the most severe effects in patients infected by SARS-CoV-2.

This type of tool allows to automate processes and a quick analysis of the results as there is sufficient data available and retraining the models. This is key to react to critical cases such as the SARS-CoV-2 virus or other existing diseases. In addition, with the appearance of new strains, such as the Brazilian, the English or Indian variant, it allows us to compare them and train specific models for those strains or specific diseases, improving the efficiency and the extraction of knowledge as well as reducing the impact of the disease on patients.

Regarding future works, there are relevant challenges such as obtaining and unifying data from different hospitals and/or countries to replicate of the model with data from other populations. This will allow to perform an external verification with the aim of verifying if the conclusions of this paper can be extrapolated to other populations. This, in turn, raises the problem of data integration from the different systems, since over the years the coding of the international disease system has evolved. However, this information is not necessarily up to date in the information systems of all hospitals across the globe. Furthermore, in order to improve the results of the presented model, more patient data could be used and/or laboratory data. This would allow not only to improve the results of this model, but also to perform other tasks such as predicting those patients who will be admitted to the ICU.

## Supplementary Information

Supplementary Tables.

## Data Availability

The data that support the findings of this study are available from the Medical Research Institute of Hospital La Fe but restrictions apply to the availability of these data, due to the nature of data which were used after signing a data processing agreement that complies with the requirements of the current legal framework in relation to data processing for the current study, and so are not publicly available. Data pseudo-anonymised are however available from the Medical Research Institute of Hospital La Fe upon reasonable request to any researcher wishing to use them for non-commercial purposes and who could guarantee and demonstrate compliance with national and European legal requirements regarding data protection. Researchers who wish to obtain a copy of the data submit their request to valdivieso_ber@gva.es.
