# Supplementary material for: Machine learning model from a Spanish cohort for prediction of SARS-COV-2 mortality risk and critical patients
Source: Sci Rep. 2022 Apr 6;12:5723. doi: 10.1038/s41598-022-09613-y (PMC8986770; doi:10.1038/s41598-022-09613-y)
Supplement: Supplementary file 1 — Supplementary Tables. [file 41598_2022_9613_MOESM1_ESM.docx]

Machine learning model from a Spanish cohort for prediction of SARS-COV-2 mortality risk and critical patients

# Reina Reina1,2*, J.M. Barrera1,2, B. Valdivieso3, María-Eugenia Gas3, A. Maté1,2, J.C. Trujillo1,2

1Lucentia dpt. Of Software and Computing Systems, University of Alicante, Carretera San Vicente del Raspeig s/n, Alicante 03690, Spain

2Lucentia lab, Av. Pintor Pérez Gil, 16, 03540 Alicante

3The University and Polytechnic La Fe Hospital of Valencia, Avenida Fernando Abril Martorell, 106 Torre H 7ª planta. 46026 Valencia Spain

*[alejandro.reina@ua.es](mailto:alejandro.reina@ua.es)

# Supplementary material

| **Variables** | **Description of variable** | **Coefficient** | **Increased**  **probability of exitus** |
| --- | --- | --- | --- |
| 496 | Chronic airway obstruction, not elsewhere classified | 1.910 | 575% |
| Age | Age of patient | 1.876 | 145% (each 10 years of patients) |
| 518.81 | Acute respiratory failure | 1.814 | 513% |
| 294.10 | Dementia in conditions classified elsewhere without behavioral disturbance | 1.811 | 512% |
| V41.9 | Unspecified problem with special functions | 1.780 | 493% |
| 588.81 | Secondary hyperparathyroidism (of renal origin) | 1.771 | 488% |
| 293.1 | Subacute delirium | 1.466 | 333% |
| 427.31 | Atrial fibrillation | 1.462 | 331% |
| 518.82 | Other pulmonary insufficiency, not elsewhere classified | 1.455 | 328% |
| 196.1 | Secondary and unspecified malignant neoplasm of intrathoracic lymph nodes | 1.281 | 260% |
| 715.96 | Osteoarthrosis, unspecified whether generalized or localized, lower leg | 1.268 | 255% |
| 310.1 | Personality change due to conditions classified elsewhere | 1.215 | 237% |
| 369.9 | Unspecified visual loss | 1.167 | 221% |
| 480.8 | Pneumonia due to other virus not elsewhere classified | 1.107 | 203% |
| 198.3 | Secondary malignant neoplasm of brain and spinal cord | 1.087 | 197% |

***Supplementary table S1.*** Top variables predicting mortality and their impact on the prediction of the best resulting model. Codes are in ICD-9-CM.

| **Iteration 1** | | **Iteration 2** | | **Iteration 3** | | **Iteration 4** | | **Iteration 5** | |
| --- | --- | --- | --- | --- | --- | --- | --- | --- | --- |
| Q | p-value | Q | p-value | Q | p-value | Q | p-value | Q | p-value |
| 200.779 | 0.000 | 478.086 | 0.000 | 367.481 | 0.000 | 365.132 | 0.000 | 348.376 | 0.000 |

***Supplementary table S2.*** Cochran’s Q test on each of the stratified k-fold cross validation.

| **Iteration 1** | | **Iteration 2** | | **Iteration 3** | | **Iteration 4** | | **Iteration 5** | |
| --- | --- | --- | --- | --- | --- | --- | --- | --- | --- |
| Q | p-value | Q | p-value | Q | p-value | Q | p-value | Q | p-value |
| 10.182 | 0.252 | 13.627 | 0.092 | 18.234 | 0.020 | 6.923 | 0.545 | 2.734 | 0.950 |

***Supplementary table S3.*** Cochran’s Q test on each of the stratified k-fold cross validation without gaussian naive bayes.
